# Complete genome sequences of two verona integron-encoded metallo-ß-lactamase (VIM)-producing enterobacterales isolated from dogs in the United States

**DOI:** 10.1128/mra.00892-24

**Published:** 2025-02-05

**Authors:** Dhruv Desai, Stephen D. Cole

**Affiliations:** 1 Department of Pathobiology, School of Veterinary Medicine, University of Pennsylvania, Philadelphia, USA; Rochester Institute of Technology, Rochester, New York, USA

**Keywords:** carbapenemase, Dogs, veterinary microbiology, carbapenem-resistance, whole genome sequencing

## Abstract

This announcement reports the complete genome sequences, created by combined Illumina and Oxford Nanopore sequencing, of two carbapenemase-producing Enterobacterales (*Escherichia coli* [LaAc-1–20] and *Enterobacter hormaechei*, strain 19632–21) isolated from dogs in the United States. Both isolates harbor a blaVIM-4 gene found on a 47 kb plasmid and on the bacterial chromosome, respectively.

## ANNOUNCEMENT

Carbapenemase-producing Enterobacterales (CPE) pose a significant risk because of their broad resistance to antimicrobial drugs (1). Verona integron-mediated metallo-ß-lactamases (VIM) are particularly significant because they are not affected by inhibitor combinations (2–4). The emergence of CPE among pets is concerning for veterinary and public health (5, 6). We performed whole genome sequencing (WGS) of two *bla*_VIM_-harboring Enterobacterales isolates from canine specimens submitted to a diagnostic laboratory as part of routine clinical care, which is exempt from IACUC approval. *Escherichia coli* (LaAC-1–20) was isolated from the feces of an 8-year-old female dog with a history of a CPE urinary tract infection using a chromogenic agar medium (ChromID Carba Agar, bioMerieux, Marcy L’Etoile, FR) and *Enterobacter hormaechei* (19632–21) isolated from a 10-year-old male dog with a blood stream infection via blood culture (Bactec Aerobic, Becton-Dickinson, Franklin Lakes, NJ) with subculture to tryptic soy agar with 5% sheep blood (TSAB) (Remel, Lenexa, KS). Antimicrobial susceptibility testing was performed on the Vitek 2 (AST-GN98 card) according to the manufacturer and interpreted according to Clinical and Laboratory Standards Institute (7). Isolates were phenotypically confirmed to produce a carbapenemase (8).

Bacteria were cultivated on under ambient conditions at 35°C. DNA was extracted using the DNeasy Blood and Tissue kit (Qiagen, Hilden, NJ) with the gram-negative bacteria protocol. Hybrid WGS was performed at a fee-for-service laboratory (SeqCenter, Pittsburgh, PA). Short-read sequencing was performed on libraries generated using Illumina DNA Prep Kit and IDT 10 bp UDI indices on the Illumina NextSeq 2000 platform generating 2 × 151 bp reads. Total reads were 5,998,832 (LaAc-1–20) and 6,116,380 (19632–21). For long read, libraries were prepared using the Oxford Nanopore Technologies (ONT) Genomic DNA by Ligation Kit (SQK-LSK109) according to the manufacturer’s protocols without shearing or size-selecting. Nanopore R9 flow cells (R9.4.1) were used with the MinION platform. The number of reads was 930,801 and 235,955 for LaAC-20–1 and 19632–21, respectively, with the high-accuracy basecalling mode of Guppy 5.0.16. Quality control and adaptor trimming were conducted using bcl-convert for Illumina and Porechop (0.2.3_seqan2.1.1) for ONT sequences. Unicycler (v.0.5.0) software was used to perform a circular hybrid assembly with ends determined and trimmed by miniasm on long and anchor reads and rotated around *dnaA* by Racon. QUAST (v.5.1.0) was used for assembly statistics (N50 = 4,819,506, N50 = 4,834,862, respectively) and annotation performed by Prokka (v.1.14.5) (9–11) in circular notation mode. Bacterial species identification was confirmed via Kmerfinder (v.1.2) (12). Default parameters were used unless otherwise noted.

Table 1 outlines the genome characteristics. Genome coverage for LaAc-20–1 and 19632–21 was 160× and 173×, respectively. Both isolates were high-risk carbapenem-resistant lineages (ST167 and ST78) by *in silico* multilocus sequence typing (v.2.0) (13–15). The *bla*_VIM-4_ gene was present on the chromosome 19632–21 but was found on a 47 kb plasmid of LaAc-20–1.

**TABLE 1:** Genome characteristics of the isolates included in this announcement.

Isolate	LaAc-1–20	19632–21
Bacterial species	*Escherichia coli*	*Enterobacter hormaechei*
Chromosome size (bp)	4,819,506	4,834,862
Number of genes	5,077	4,899
Protein coding sequences	4,708	4,785
Number of plasmids	11	5
Range of plasmid sizes (bp)	2,064–114,629	2,279–135,804
GC content (%)	50.68	55.14

## Data Availability

Data have been deposited in GenBank under the BioProject accession no. PRJNA1144140. The genomes at JBGEDN010000000 and JBGEDO000000000. The 47 kb plasmid of LaAc-20-1 is at JBGEDN010000004.1. Raw sequence reads are at SRX25596228 and SRX25596226 for LaAc-20-1 SRX25596229 and SRX25596227 for 19632-21.
